# Supplementary material for: A novel approach to digital characterisation of Tertiary Lymphoid Structures in colorectal cancer
Source: Front Immunol. 2025 Jan 28;16:1500792. doi: 10.3389/fimmu.2025.1500792 (PMC11827424; doi:10.3389/fimmu.2025.1500792)
Supplement: Supplementary file 1 [file DataSheet1.pdf]

Figure S1

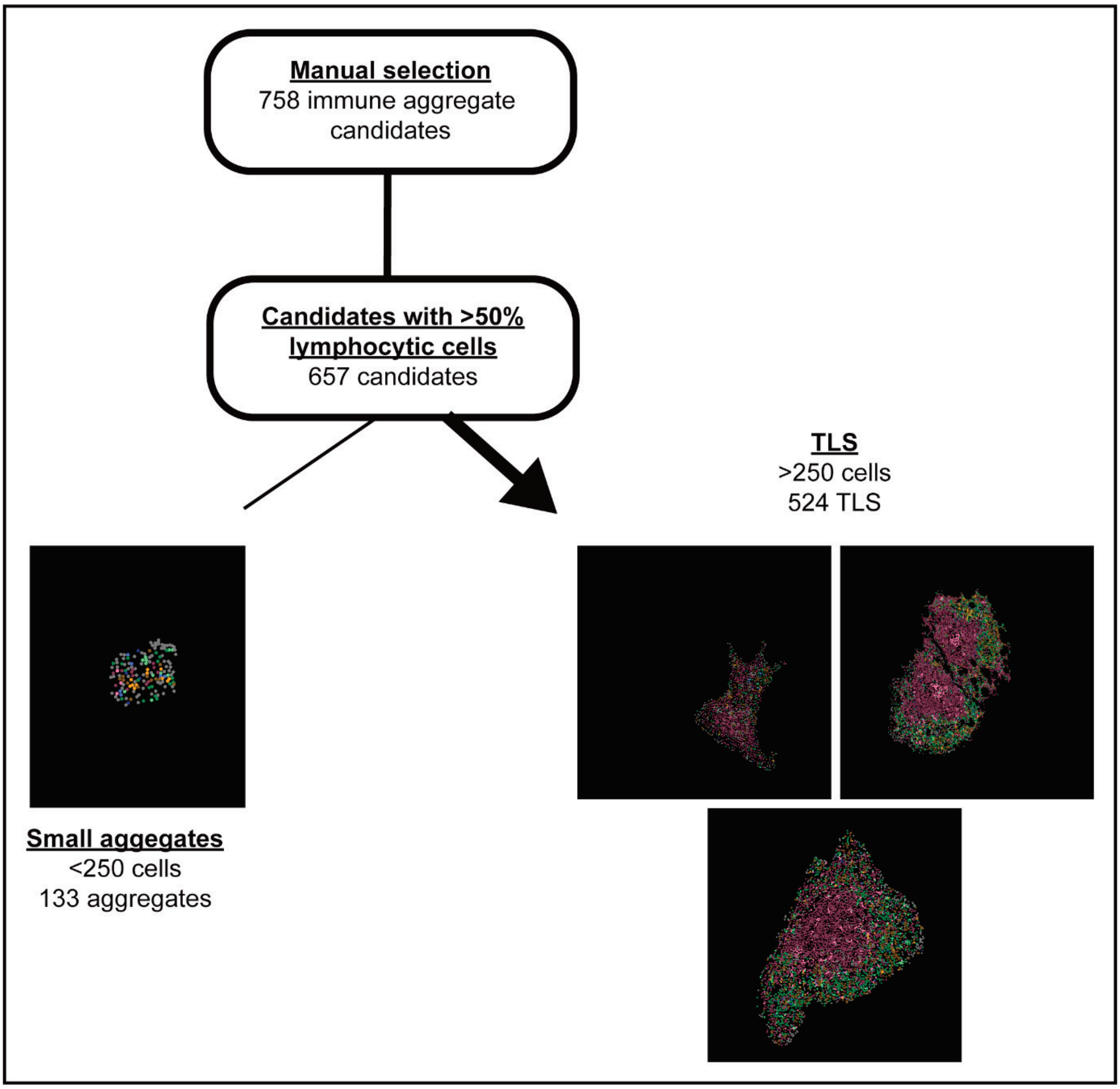

Flowchart of TLS analysis. All lymphocyte clusters with potential to be part of TLS were selected within regions of interest for cell segmentation, phenotyping and digital analysis, as described in the Methods. Clusters comprising ≥50% non-lymphocytic cells ("undefined" cells), were first removed from further analysis, followed by clusters comprising less than 250 cells.

Figure S2

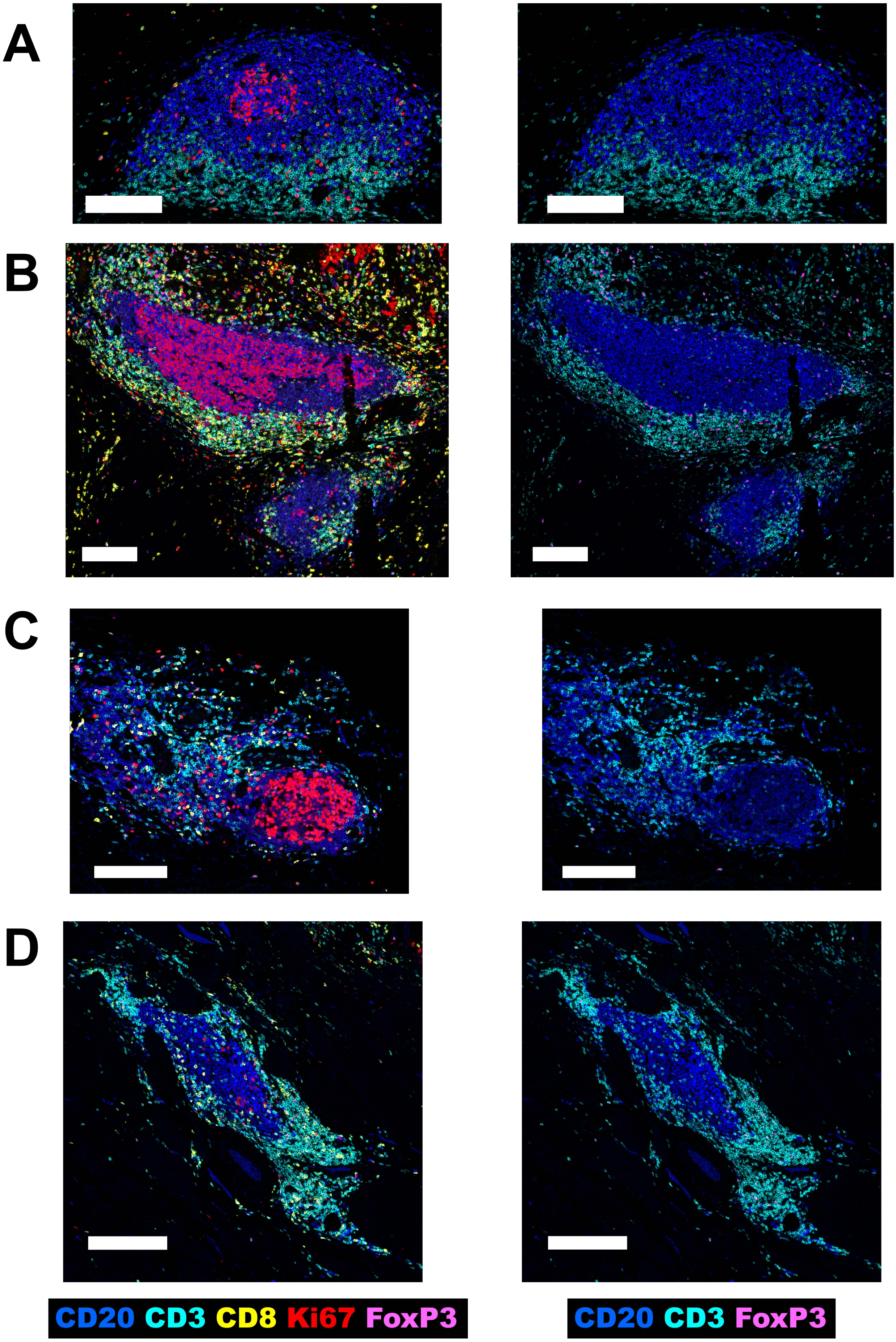

E

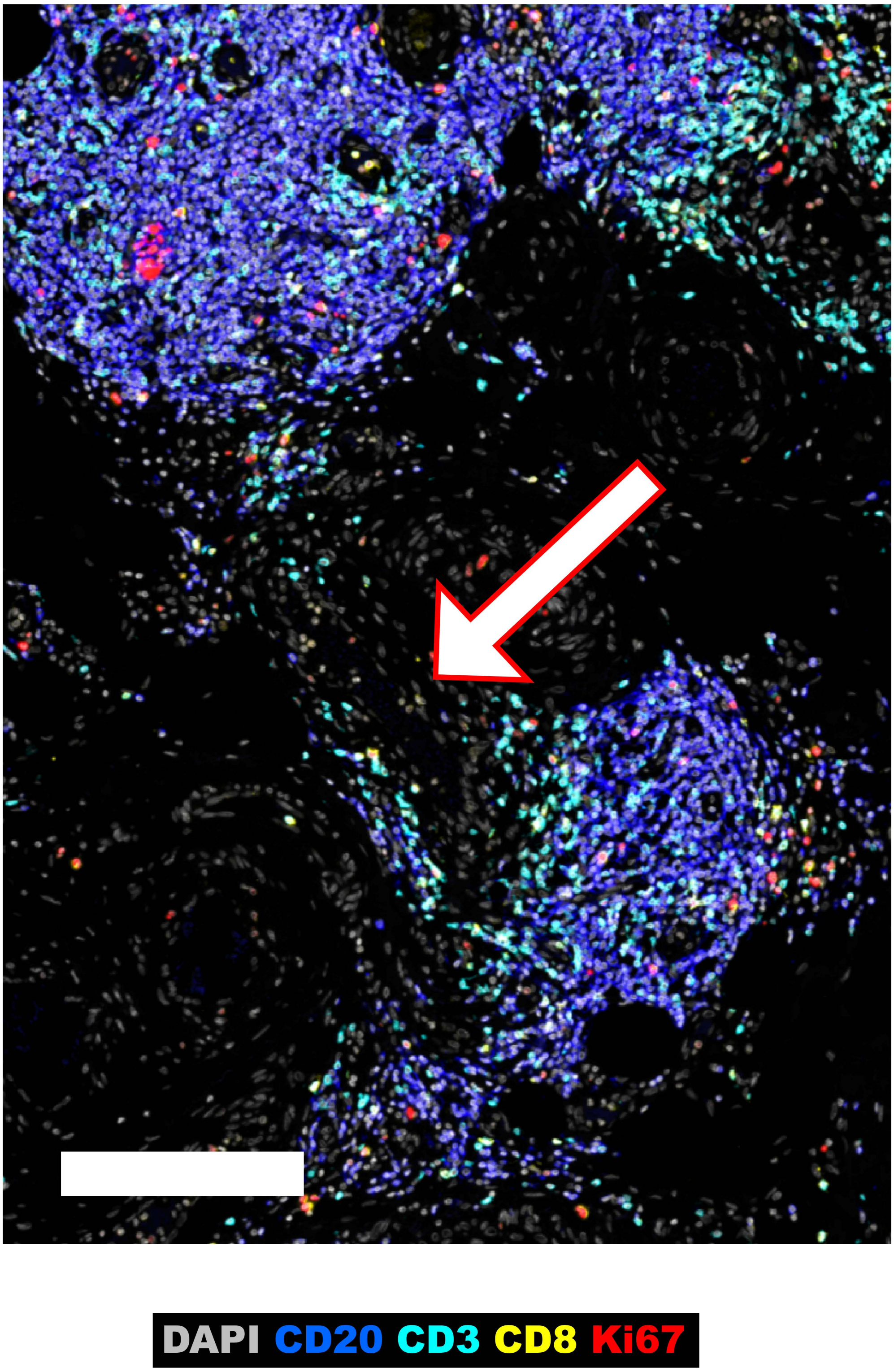

Foxp3 staining in CRC TLS and connecting interfollicular lymphocyte strands. (A-D) Examples of CRC TLS from Figure 2A-D are shown with CD20 (blue), CD3 (cyan),CD8 (yellow), Ki67 (red) and FoxP3 (magenta) on the left. On the right, the same images with just CD20, CD3 and FoxP3. **(E)** A closeup of the strand containing lymphocytic cells connecting two follicles seen in Figure 2J. Scale bars are 200µm.

Figure S3

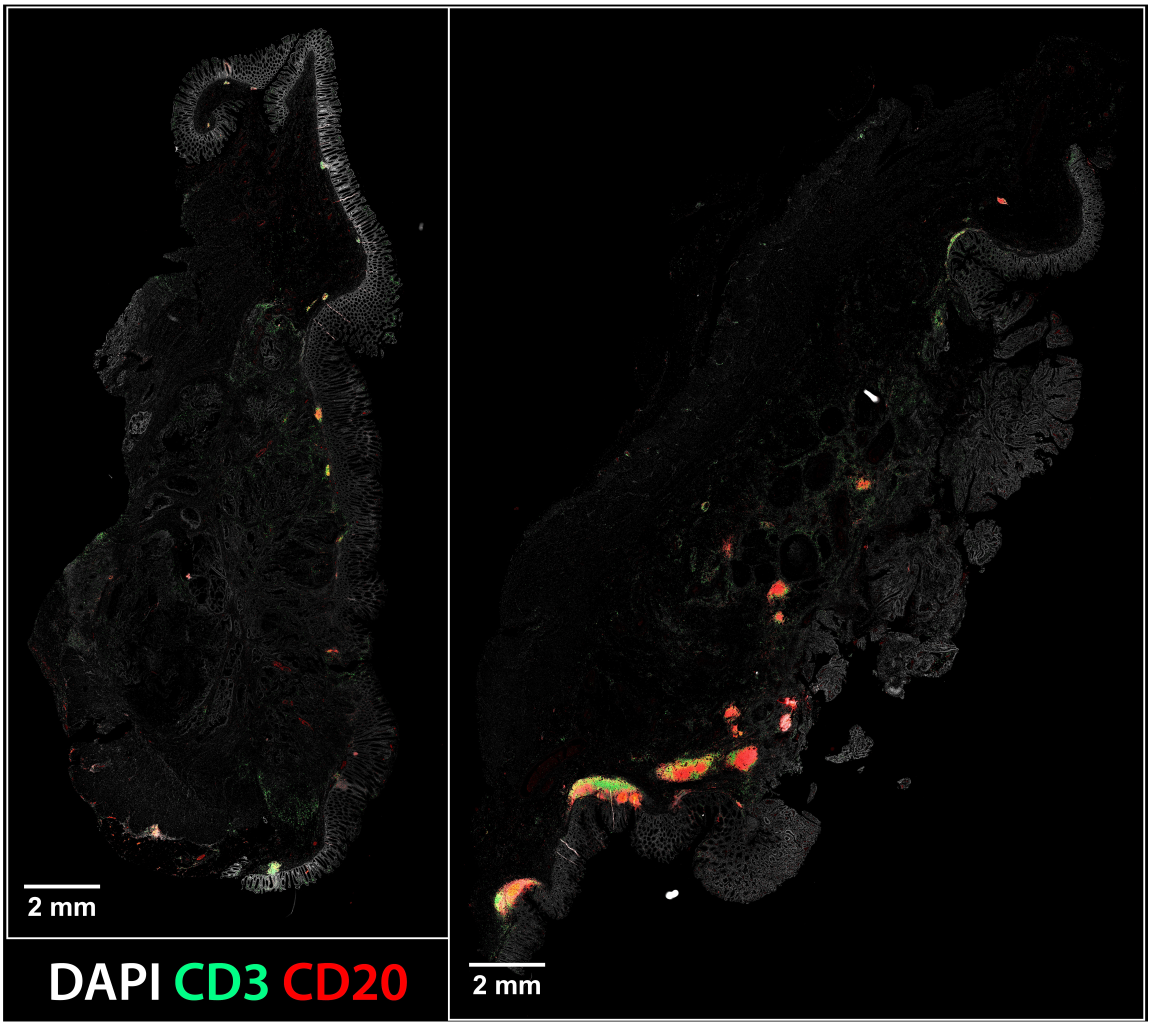

Disparity between the count of TLS domains and total area of TLS. Whole scan images of tissue sections from P006 (left) and P013 (right), showing only DAPI (light grey), CD3 (green) and CD20 (red).

Figure S4

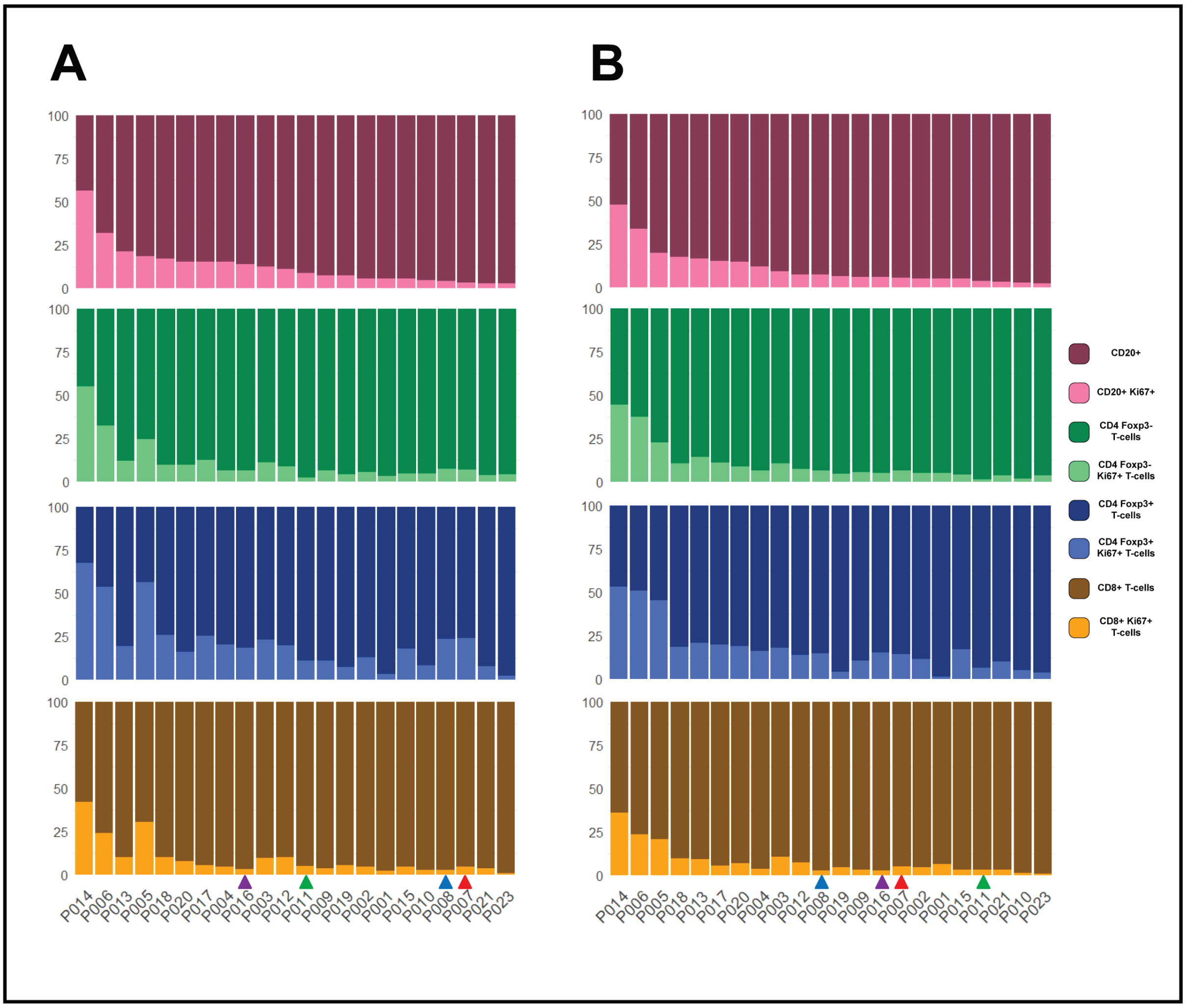

Rankings of patients by quantification of B-cell Ki-67 in individual TLS domains vs all TLS domains combined. 4 lymphocyte subsets were identified within TLS domains and the proportions of Ki-67+ cells within those subsets were calculated and plotted in stacked plots. Patients were ranked according to the proportion of B-cells expressing Ki-67, calculated according to two different methods. **(A)** As for Figure 3B and 3C, these proportions were calculated for each individual TLS domain then averaged. **(B)** As for Figure 6A, cell phenotypes were quantified across all pooled TLS domains within the tissue sample. Coloured arrows are used to highlight four patients (P007=red, P008=blue, P011=green, P016=purple) who had significant changes in rank between the two different approaches.
